# Facilitators and barriers perceived by health professionals in the implementation of Managing Cancer and Living Meaningfully (CALM) psychotherapy in Santiago

**DOI:** 10.3332/ecancer.2021.1256

**Published:** 2021-06-30

**Authors:** Loreto Fernández-González, Moisés Russo Namías, Paulina Bravo

**Affiliations:** 1 Instituto Oncológico Fundación Arturo López Pérez, José Manuel Infante 805, Santiago 750000, Chile; 2 Dalla Lana School of Public Health, University of Toronto, 155 College St, Toronto, Ontario M5T 3M7, Canada; 3 Global Institute of Psychosocial, Palliative and End of Life Care, 700 Bay Street, Suite 2303, Toronto, Ontario M5G 1Z6, Canada; 4 Escuela de Enfermería, Pontificia Universidad Católica de Chile, Av Vicuña Mackenna 4860, Santiago 7820436, Chile; 5 Facultad de Medicina, Universidad Diego Portales, Av. Manuel Rodríguez Sur 415, Santiago 8320000, Chile; 6 Centro Núcleo Milenio Autoridad y Asimetrías de Poder, Santiago 8320000, Chile; a https://orcid.org/0000-0001-5026-6438; b https://orcid.org/0000-0003-0944-5244; c https://orcid.org/0000-0001-7378-6487

**Keywords:** palliative care, Chile, psychotherapy, education, implementation science

## Abstract

**Introduction:**

Palliative care (PC) for advanced cancer is guaranteed by law in Chile, but the formal training for it is insufficient. Training models have emerged internationally that enable professionals to be better prepared for the provision of psychotherapy in PC. The objective of this study is to explore health professionals’ perceptions of the ‘Managing Cancer and Living Meaningfully’ (CALM) psychotherapy and the perceived barriers and facilitators to its implementation, based on a theoretical training.

**Methods:**

A qualitative study was carried out with health professionals working in oncology and/or PC and participating in a CALM training. A focus group was conducted after to explore the experience of CALM training and the perceived barriers and facilitators to its implementation. A thematic analysis of the content and an analysis of the facilitators and barriers to the implementation of mental health services were carried out.

**Results:**

Twenty four professionals participated in the training, six of whom were part of the subsequent focus group. There was a consensus that the training was a positive professional experience and that it is a culturally sensitive and feasible intervention for application in Chile. The barriers identified include institutional bureaucracy as resistance to change, the excess workload of the clinical teams and the absence of spaces for more in-depth training.

**Conclusions:**

CALM is a useful and relevant framework for the training of health professionals working in oncology and PC. In Chile, there is a need for training spaces on this topic. Future research and organisational studies should evaluate professionals’ beliefs about, and resistance to, adopting evidence-based psychotherapeutic interventions.

## Introduction

Palliative care (PC) aims to improve quality of life and alleviate the suffering of patients and family members of those who have an advanced or potentially life-threatening disease [1]. The approach to this suffering is not limited just to physical symptoms, but also includes an assessment and treatment of psychosocial and spiritual aspects [2]. It is, therefore, defined as a multi-professional discipline that requires specially-trained teams. However, even though its provision is considered by the World Health Organization (WHO) as a human right, the same entity states that one of the most significant barriers to its correct implementation is the lack of professionals with specific training in PC [1].

Likewise, although there is a consensus that providing psychosocial care to patients with advanced or metastatic cancer should be part of the standard of care, there is a significant gap in access to it: not just because of a lack of trained staff, but also because most interventions developed in this field are relevant to high-income countries [3]. This represents an access gap for professionals in the Global South due to language and cultural barriers to adapt such interventions in local contexts. Addressing this gap is key as 78% of people who require PC live in low- and middle-income countries, according to WHO data [1].

In Chile, access to PC in both the public and the private health system is guaranteed by law. Also, the corresponding national clinical guideline stipulates the multidisciplinary composition of health care teams and mentions psychological care as a service available to patients [4]. However, the guideline lacks recommendations on how to conduct psychological assessments and what content to address in the clinical work with this population. This constitutes a gap in clinical practice and reflects the absence of the incorporation of evidence-based interventions.

There are currently several short-term psychotherapies developed for the palliative context, with positive evidence about their effectiveness in reducing symptoms and improving quality of life [5]. Although they have some similarities in the issues addressed, they differ in that some have been designed for end-of-life patients or can be adapted to work with patients in group or individual settings. One of these interventions is Managing Cancer and Living Meaningfully (CALM), a short-term semi-structured psychotherapy for the patient and his/her primary carer, (which) addresses the issues of living with advanced cancer [6]. Developed in Canada, CALM has proved to be culturally adaptable to other countries and has been promoted as a feasible intervention to be implemented in different contexts and by professionals from different disciplines [7].

The objective of this study was to explore health professionals’ perceptions of the ‘Managing Cancer and Living Meaningfully’ (CALM) psychotherapy and the perceived barriers and facilitators to its implementation, based on a theoretical workshop.

## Materials and methods

As part of the implementation of a pilot training programme in CALM, a qualitative study was conducted with some of the health professionals who received this training.

### CALM

CALM is a short-term, evidence-based, individual-format psychotherapy of three to six sessions, in which topics relevant to patients are discussed. It may include the primary caregiver in more than one session. These include four domains specific to living with advanced cancer, including: management of symptoms, interpersonal relationships, sense of purpose and reflection on mortality. It is designed to be provided during a 3-to-6-month course, with the possibility of further sessions where appropriate. Its target population consists of patients with advanced or metastatic cancer who have a minimum prognosis of 6 months. The CALM therapist can have training in different disciplines related to PC. The active supervision of clinical cases by therapists trained in this psychotherapy is encouraged to ensure the integrity of the intervention. Its results have been effective in reducing depressive symptoms and better end-of-life preparation [6].

### Training structure

The project was developed as a quality improvement project at a non-profit private cancer centre in Santiago. Health professionals who work in oncology and/or PC were invited to participate in the training. The training was given over 3 monthly sessions of 3 hours long. The original material from CALM

workshops was used. The content of the training consisted of reviewing the theoretical foundations of CALM, setting characteristics, and typical format of the intervention. In addition, subtitled videos of CALM sessions with real patients were reviewed to exemplify concepts and observe the therapist’s interventions. All training was given by a professional psychotherapist with extensive training and clinical experience in CALM. All participants signed a confidentiality agreement following the original guidelines for safeguarding the privacy of videotaped patients, and an informed consent for the dissemination of results.

### Post-training focus group

After the training, participants were invited to a focus group to explore the CALM training experience and the perceived barriers and facilitators to its implementation. This was recorded and transcribed for further analysis. A thematic analysis of the results was performed and coded for the subsequent creation of categories [8]. Results were organised following Proctor *et al* [9], on the implementation of mental health services in organisations. This model groups the following outcomes: feasibility, fidelity, penetration, acceptability, sustainability, escalation and costs.

## Results

### Training and participants

Three training sessions were given between May 2018 and July 2018. Twenty-four health professionals working in public and private health centres in Santiago participated. Most of them identified themselves as women (87.5%). The most common professional backgrounds were psychology (45.8%) and psychiatry (25%). Most reported working with cancer patients full-time. 37.5% reported lacking specialized training on the subject (Table 1).

### Focus group results

A focus group was held one month after the last training session. Six professionals participated, all of whom were women, from four centres (two of which were public and the other two private). Of these, two were psychologists, two nurses and two psychiatrists. The categories identified in the analysis are presented below.

**1. Appraisal of CALM: **CALM was perceived as a useful tool for the psychosocial management of the palliative patient, and a culturally sensitive intervention, which can be adapted to the local needs of patients and teams. In addition, the conceptual organisation in domains was perceived as a relevant model, comprising the main foundations of psychotherapeutic work with advanced cancer patients, and was seen as especially helpful in disseminating their professional work in their respective institutions and strengthening their role as therapists.

*‘There are some concepts that have stayed with me and I see them, I read them when I see the patients (...). It’s like being able to give it a name and say “of course, this happens to a lot of people. Someone described it, they observed it and many people have observed it and described it, so it happens”.’*

*‘Something that I have put into practice more are the domains: it gives me the confidence to contact other teams. I know that it sounds obvious, but it’s not always done’.*

*‘Everything that shortens the journey in a good way, so that you have more clarity and are less burdened, I think it helps to support the therapist as well’.*

**2. Experience of CALM training: **There was a consensus among participants in the focus group that the training was a positive professional experience. In terms of the methodology of the training, the value of the video sessions with real patients was highlighted as especially useful in understanding the intervention both in theory and in practice. However, the participants mentioned that the training was too introductory (‘just a glimpse’) to enable them to practice the psychotherapy themselves. In this regard, although they felt motivated to continue to delve into CALM, this enthusiasm was tempered by a perceived lack of continuity in the training.

*‘I didn’t know anything anything about it before, so I [got] the feeling that it was a general introduction like... like knowing what it’s about rather than, more than, knowing how to apply it’.*

*‘I would love to be able to apply it or to be able to start putting something together to pseudo-apply it, [although] I wouldn’t feel able to do it just like that, or like in that video we saw... wonderful... but it did... it made me want to keep coming back, it made me want to keep learning...’*

*‘We need someone to keep training us... And I don’t know if in Canada there is more formal, more structured training where they give you a diploma and say, “you are qualified”.’*

**3. Implementation of CALM: **On being consulted about how they would implement CALM in their workplaces, the professionals identified that, together with continuing the training, disseminating the intervention in their respective centres (was) the first step. However, they mentioned that this might be hindered by the excessive burden of work among the clinical teams, and especially among the PC unit teams in their institutions. Furthermore, organisational bureaucracy (expressed as resistance to change) may hinder the implementation of CALM in its clinical and academic research dimensions.

*‘I think there is a lot of resistance to change, but also a lack of protected time for training activities... because, for example, the palliative care team at our hospital is completely overstretched, so proposing that they come in on a Saturday morning was like (...) ... “no”.’*

*‘I can get there and do it, but that the institution says, “we do CALM here”, something like that? I find it very difficult... I would have to talk to the psychiatrist, to the palliative [team], to the academic (...). I don’t think it would be easy’.*

*‘I think it is very important to have these initiatives and fortunately we have a very good vibe and communication between us, but of course to expand on something, to do something more... formal, you don’t have to deal with the palliative team, but with the institution...’*

**4. Analysis according to implementation outcomes: **By analysing the categories mentioned previously, it is possible to group them according to the Proctor *et al* [9] model, identifying them as facilitators or barriers to the implementation of CALM as a mental health intervention. In particular, there was a positive perception of the feasibility, acceptability and fidelity of adopting the intervention. In contrast, escalation and sustainability would constitute barriers to its adoption as a standard of care in health centres. Table 2 describes how the outcomes defined in this model are grouped as facilitators or barriers.

## Discussion

This study shows the results of a training experience among Chilean professionals in CALM psychotherapy in Santiago, identifying potential facilitators and barriers to its implementation. The results presented are useful in understanding the difficulties and opportunities when it comes to implementing evidence-based interventions in the field of PC in the country.

Firstly, it was observed that Chilean professionals (reported) a lack of specialised training in PC and the psychosocial management of the palliative patient. Although the majority reported working with oncology patients on a full-time basis, more than a third of the participants reported not having a specialised degree or training on the subject. This is consistent with what the WHO reports and other authors and reflects the gap between PC needs and the lack of specialised professionals in the area at a global and regional level [1, 10, 11]. These results are particularly worrying as Chile is considered a high-income country where access to PC is guaranteed by law. Thus, our results shed light on the gaps in the training of human capital, not just within the country but probably also across Latin America (as a whole).

Our results show the local interest in acquiring tools and knowledge in mental health interventions for the PC patient, specifically CALM. This short-term, evidence-based psychotherapy of Canadian origin has proved to be effective as a non-pharmacological intervention for depressive symptomatology in patients with advanced cancer. Its effectiveness and flexibility in adapting to diverse populations has led to global interest in its adoption and implementation in several countries, with positive reception and implementation experiences [12, 13]. In this (respect), the training carried out in Chile constitutes a milestone in Latin America in the dissemination of this intervention, (as it is) the first Spanish-speaking country to hold this training. This reflects not only an effort to overcome language barriers by translating all the original material, but also constitutes a successful collaboration exercise between countries from the Global North and the Global South in the development of collaborative networks and professional training [14, 15].

The emotional and psychosocial needs of patients with advanced cancer are highly challenging for treatment teams since they are multidimensional and dynamic over time. The clinical presentation of symptoms may differ from psychiatric settings in the non-cancer population, and thus may be difficult to identify and manage [16]. Our results show that the CALM training provided attendees with tools to identify these manifestations in Chilean patients. Likewise, the professionals reported that they felt more confidence and security in their ability to do their jobs, and that having the CALM framework contributed to this perception of self-sufficiency. This is consistent with other work in oncology and PC and shows that training in this area not only has a positive impact on patients, but also on providers [17, 18]. In this respect, CALM is perceived as a beneficial tool for clinical work, which would facilitate its implementation.

As for the barriers perceived by the professionals, they highlight institutional aspects and elements of the organisational culture of PC units. These barriers are not specific to CALM but are common to interventions of this type. The difficulties for the integration and implementation of psychosocial services as a standard of care in oncology and PC have been widely documented in the literature [19] and point to the need for a paradigm shift from disease-centred management to one focused on the comprehensive care of cancer patients, including (those with) advanced cancer [14, 20].

The present study has strengths and limitations. Among its strengths is the novelty of the intervention and the fact that it was the first local training event in the region in collaboration with the Canadian CALM team. The positive reception and experience of the attending professionals sets a precedent for future collaborations between national and international centres both in the creation of continuing education opportunities and in the piloting and implementation of evidence-based strategies and interventions in the field of PC. With regard to the limitations, the training was only attended by professionals from Santiago, and the fact that it was held on Saturdays may have affected the attendance of professionals interested in participating. Furthermore, it was introductory in nature, so it did not constitute an official certification in CALM. However, the participants showed an interest in continuing their training, and the success of this initiative suggests its possible resumption or repetition in the future. Methodologically, only one focus group was held, which may have affected its qualitative thoroughness. However, the participants in the focus group varied in their professional training and in the centres in which they worked, which added richness to the perspectives presented in the group discussion.

## Conclusions

In conclusion, CALM is a short-term psychotherapy for patients with advanced cancer, which framework and content were perceived as useful and relevant by the health professionals who participated in this initiative. There is a need for PC training spaces in the country and it is crucial to have training opportunities and to disseminate effective interventions that are culturally sensitive to different populations. Future research and organisational studies should evaluate PC professionals’ beliefs about, and resistance to, adopting evidence-based psychosocial interventions.

## Funding statement

Financial support was received from the National Fund for Research and Development in Health (FONIS, according to its Spanish acronym) (No SA18I0058) for the writing of this article. FONIS had no influence over the design, collection or analysis of the data, or over the preparation or approval of the current manuscript.

## Conflicts of interest

None of the authors declare any conflicts of interest.

## Figures and Tables

**Table 1.** Description of training participants.

		Participants *n* = 24
Gender (%)		
	Female	87.5
	Male	12.5
Age (average, range)		38.7 (26–65)
Occupation^a^ (%)		
	Psychologist	45.8
	Psychiatrist	25.0
	Art therapist	16.7
	Nurse	8.3
	Physician	4.2
	Family counsellor	4.2
	Social worker	4.2
Workplace (%)		
	Public health centre	41.6
	Private health centre	58.4
Years of experience (average, range)		4.6 (0–30)
Weekly hours (average, range)		30.5 (10–44)
Specialized training (%)		
	Postgraduate degree	25.0
	Courses	25.0
	Medical specialty	12.5
	Internships	12.5
	None	37.5
	No answer	4.2
English proficiency level (%)		
	Basic	33.3
	Intermediate	45.8
	Advanced	16.7
	No answer	4.2

a There are two psychologists/art therapists

**Table 2.** Facilitators and barriers to the implementation of CALM, according to the Proctor et al [9] model.

	Implementation outcomes	Participants’ perceptions
Facilitators	Acceptability	The content of CALM is a contribution to clinical practice, both for patients and therapists.
	Fidelity	There is a perceived consistency between what the intervention describes and what the professionals observe in their practice.
	Feasibility	The CALM structure may be applied by Chilean therapists.
Barriers	Penetration/escalation	There is a perceived difficulty in disseminating what has been learned, and training other professionals and teams due to time constraints and workload.
		The institutional organisation is perceived as resistant to change and to the adoption of new interventions, despite the possible interest of other professionals.
	Sustainability	Difficulty in continuing with CALM training.
